# Self-assembling biomolecules for biosensor applications

**DOI:** 10.1186/s40824-023-00466-8

**Published:** 2023-12-05

**Authors:** Ji-eun Kim, Jeon Hyeong Kang, Woo Hyun Kwon, Inseo Lee, Sang Jun Park, Chun-Ho Kim, Woo-jin Jeong, Jun Shik Choi, Kyobum Kim

**Affiliations:** 1 Department of Chemical & Biochemical Engineering, Dongguk University, Seoul, 04620 Republic of Korea; 2 Department of Biological Sciences and Bioengineering, Inha University, Incheon, 22212 Republic of Korea; 3 Laboratory of Tissue Engineering, Korea Institute of Radiological and Medical Sciences, Seoul, 01812 Republic of Korea; 4 Department of Biological Engineering, Inha University, Incheon, 22212 Republic of Korea; 5 Department of Materials Science and Engineering, Yonsei University, Seoul, 03722 Republic of Korea

**Keywords:** Molecular self-assembly, Supramolecular biosensor, Electrochemical biosensor

## Abstract

Molecular self-assembly has received considerable attention in biomedical fields as a simple and effective method for developing biomolecular nanostructures. Self-assembled nanostructures can exhibit high binding affinity and selectivity by displaying multiple ligands/receptors on their surface. In addition, the use of supramolecular structure change upon binding is an intriguing approach to generate binding signal. Therefore, many self-assembled nanostructure-based biosensors have been developed over the past decades, using various biomolecules (e.g., peptides, DNA, RNA, lipids) and their combinations with non-biological substances. In this review, we provide an overview of recent developments in the design and fabrication of self-assembling biomolecules for biosensing. Furthermore, we discuss representative electrochemical biosensing platforms which convert the biochemical reactions of those biomolecules into electrical signals (e.g., voltage, ampere, potential difference, impedance) to contribute to detect targets. This paper also highlights the successful outcomes of self-assembling biomolecules in biosensor applications and discusses the challenges that this promising technology needs to overcome for more widespread use.

**Graphical Abstract:**

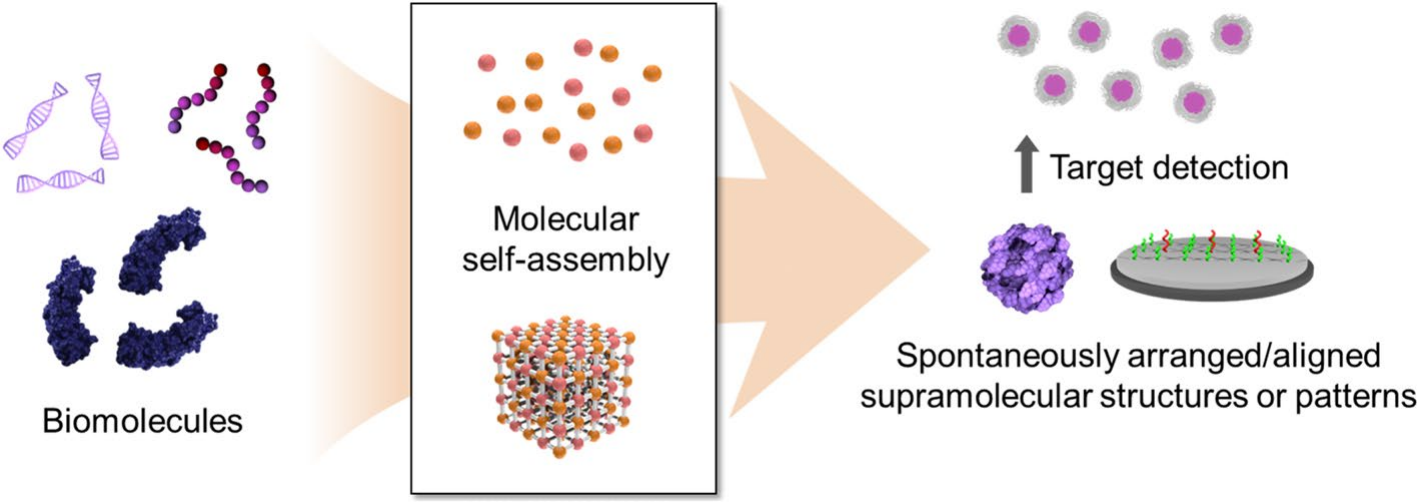

## Introduction

**Fig. 1:**
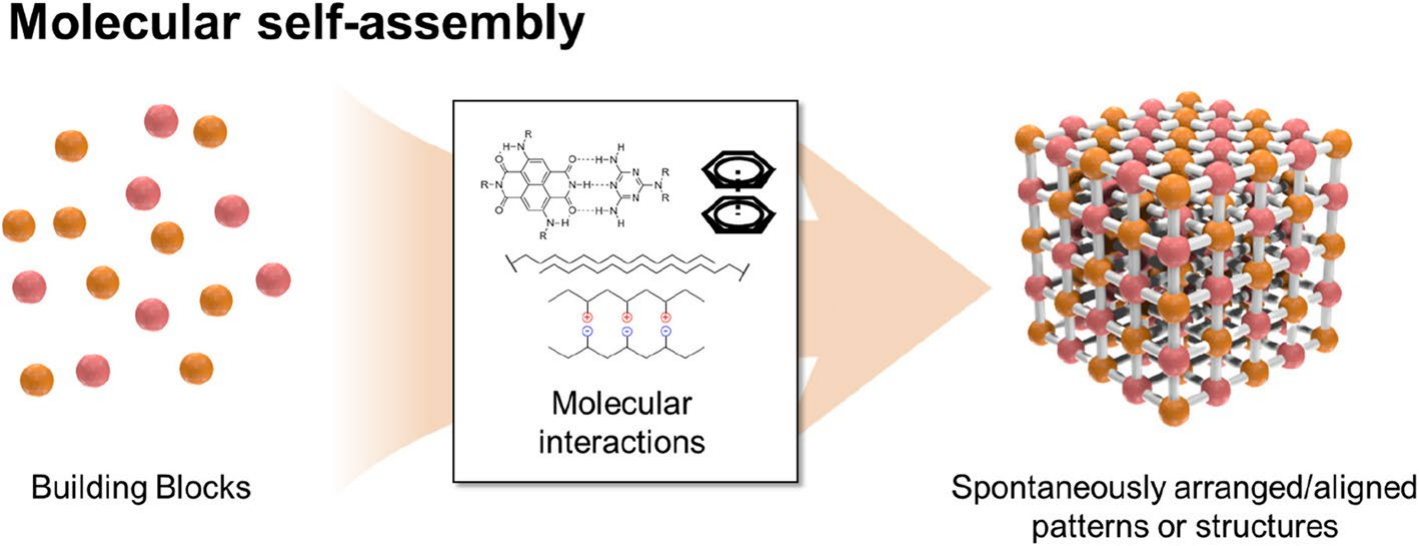
Schematic illustration of molecular self-assembly

Self-assembly has attracted great interest in the field of biotechnology as a simple and effective method for developing bioactive nanostructures. In addition, research on the self-assembly behavior of biomolecules have provided clues to understand biological phenomena, as numerous biomaterials (e.g., cells, organelles, and vesicles) are organized through the spontaneous bottom-up process [1, 2]. Self-assembly is the process of organizing highly arranged/aligned patterns or structures based on local molecular interactions between molecular components (Fig. 1), such as electrostatic associations (hydrogen, ionic, and halogen bonding), van der Waals forces (dipole-dipole, London dispersion forces), and π-interactions (π-π stacking, π-polar, π-cation, and π-anion) [3–8]. Therefore, it is possible to control physicochemical properties of self-assembled nanostructures by appropriately designing their building blocks at the atomic or molecular level [9–11].

Biomolecule-based self-assembled nanostructures (BSNs) are promising materials to be used in biosensor applications. BSNs can effectively detect targets by exposing multiple receptors on their surface, which is based on the fact that (1) the exposure of receptors in multiple directions on the nanostructure surface increases the probability of encountering target molecules and (2) multivalent interactions generally exhibit significantly higher binding affinity and selectivity than monovalent interactions [12–17]. BSNs constructed based on various noncovalent interactions can cause changes in the supramolecular structure upon target binding, which has the potential to be utilized for sensor signal generation and transduction [17]. In addition, the hybridization of self-assembling biomolecules with various non-biological substances can make BSN-based biosensors exhibit intriguing electronic, magnetic, mechanical, and optical properties [18].

However, to the best of our knowledge, there are scarcely any commercialized BSN-based biosensors currently available apart from a few examples, including Zimmer & Peacock’s sensor utilizing screen-printed electrodes for electrochemical sensing, and Metrohm DropSens’s sensor employing screen-printed electrodes for spectroelectrochemical sensing [19, 20]. These commercialized biosensors are primarily designed to detect low molecular weight compounds or simple proteins by employing a relatively simple self-assembled monolayer (SAM) structure that exposes antibodies. Consequently, they fail to fully harness the benefits derived from the diversity of biomaterials or from a supramolecular approach based on self-assembly. Additionally, the significant cost of antibodies further constrains their broader application. Thus, to promote the development of more effective and diverse BSN-based biosensors, extensive research is required, ranging from fundamental scientific principles to applied technological aspects.

This review aims to provide a comprehensive analysis of the current state of biosensor applications using BSNs, exploring where the field stands today and what future research directions need to be pursued. In detail, we introduce various types of supramolecular biosensors based on various self-assembling biomolecules (e.g., peptides, proteins, and oligonucleotides) and their properties that make them attractive for biosensing applications (Sect. 2). In addition, we examine the design and application of a voltammetric, amperometric, potentiometric, and impedimetric electrochemical biosensing platforms for disease diagnosis and monitoring using self-assembling biomolecules in Section 3. Overall, we suggest the importance of biosensor design based on a deep understanding of self-assembling biomolecules and electrochemical biosensing platforms and discuss challenges and future research directions in biosensor applications of self-assembling biomaterials.

## Supramolecular approaches in biosensing

### Peptide- and protein-based supramolecular biosensors

**Fig. 2:**
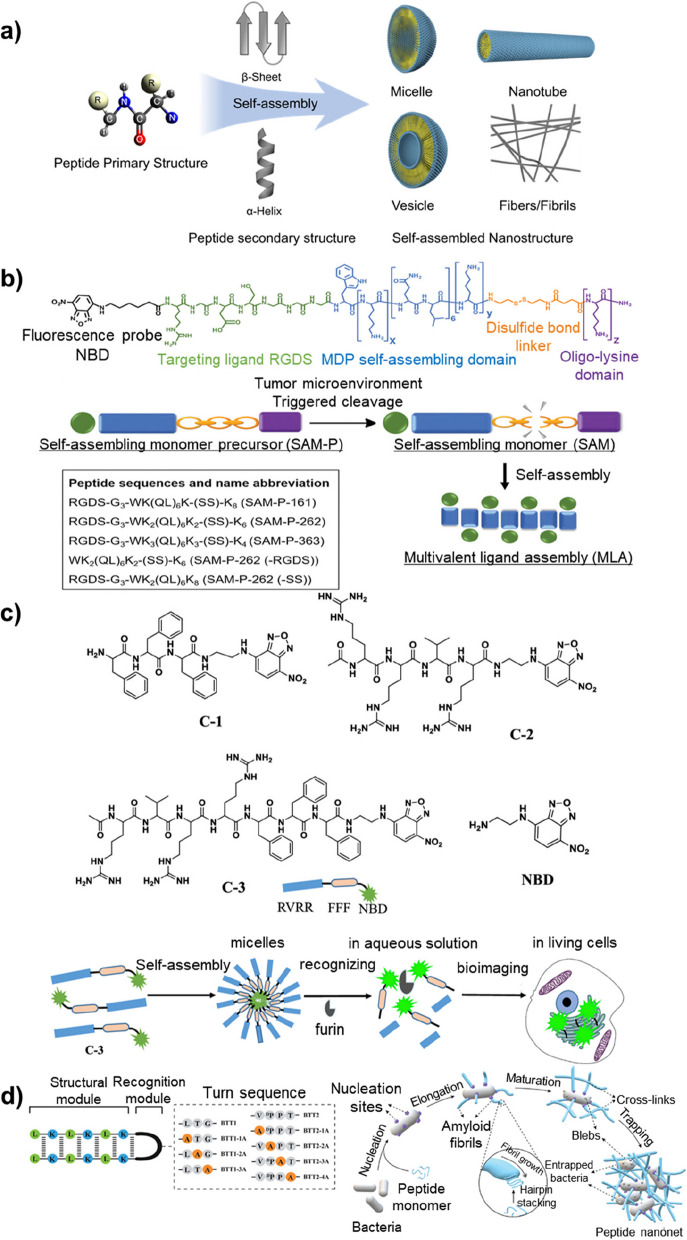
**a** The self-assembly behavior of peptides can be controlled by changing their primary (amino acid sequence) and secondary (e.g., α-helix, β-sheet, and β-hairpin) structures. **b** Schematic illustration of the chemical design of the assembling precursor that undergoes reduction-triggered self-assembly to form supramolecular assemblies with multivalent ligand presentation for tumor targeting. Reprinted with permission from ref. [33]; Copyright 2020, Wiley. **c** Schematic illustrations of the self-assembly process and furin detection. Reprinted with permission from ref. [34, 35]; Copyright 2019, American Chemical Society. **d** Schematic mechanistic pathways of bacteria-induced formation of peptide nanonets. Reprinted with permission from ref. [34, 36]; Copyright 2022, Wiley

Peptides can be synthesized with countless amino acid combinations and molecular structures through solid-phase peptide synthesis (SPPS), thereby exhibiting diverse chemical, physical, and biological characteristics [21]. Appropriately designed peptides can form nanostructures through self-assembly, based on the combination of intra- and inter-molecular non-covalent interactions such as hydrogen bonding, electrostatic interactions, hydrophobic interactions, van der Waals forces, and π-π stacking [22, 23]. The self-assembly behavior of peptides is controllable by altering their amino acid sequences, secondary structures (e.g., α-helix, β-sheet, and β-hairpin), and environmental conditions, such as pH, temperature, and ion strength (Fig. 2 a) [24–28]. This feature is useful not only for forming sophisticated nanostructures but also for generating and transducing signals in response to external stimuli [29, 30]. Hence, peptide-based self-assembled nanostructures have been considered as promising materials for biosensors, based on their bioactivity and biocompatibility [29, 31, 32].

Designing peptides to undergo chemical transformation and subsequent self-assembly under a certain condition is a promising strategy to develop stimuli-responsive biosensors. For example, the Yang group reported the tumor-responsive hydrogel assembly of peptides, which was triggered by their interaction with alkaline phosphatase (ALP) and tumor cell membrane receptors (CCK2R) [37]. Dong et al. also developed tumor microenvironment-responsive supramolecular nanofibers using peptides including a self-assembling monomer precursor (SAM-P) domain (Fig. 2 b) [33]. At the tumor site, SAM-P underwent tumor-triggered cleavage and released its active form of β-sheet forming monomer, whereby organizing supramolecular nanofibers with different lengths. As the peptides also contained a tumor cell targeting domain (RGD), the self-assembled structures displayed the ligands in a multivalent way, which significantly enhanced their tumor cell specificity and sensitivity. On the other hand, Yi et al. developed self-assembled peptide nanostuructures that can be disassembled and exhibit fluorescent signal when recognizing target molecules (Fig. 2 c) [35]. In this study, the self-assembling peptide (C-3) was composed of the following components: a nitrobenzoxadiazole (NBD) motif as a fluorescent output reporter, a Phe-Phe-Phe tripeptide sequence contributing to hydrophobicity, an Arg-Val-Arg-Arg sequence for membrane penetration and hydrophilicity of C-3, and a substrate for the specific detection of furin, which is a proprotein convertase abnormally expressed in several cancers. In the intratumoral environment, the C-3 peptides were selectively cleaved by furin, which resulted in the disassembly of their self-assembled micelle structures and production of “turn-on” fluorescent signal. These tumor cell targeting strategies based on stimuli-responsive properties of peptides hold potential to be used in cell-specific molecular imaging and therapeutics.

Various self-assembled peptide nanoprobes functionalized with antimicrobial peptides (AMPs) were developed for bacteria detection. These probes have demonstrated rapid and accurate diagnostic capabilities, enabling the effective diagnosis and treatment of bacterial infections [38–40]. Based on studies showing that vancomycin can specifically bind to the d-Ala-d-Ala moiety on gram-positive bacterial cell walls [41], Yang et al. reported the surface-induced self-assembly of peptide-vancomycin conjugates, which enabled the detection of bacteria with the aid of environment-sensitive fluorescent probes [36]. Interestingly, the peptide self-assembly also resulted in the bacterial inhibition, based on the trap-and-kill mechanism. Trap-and-kill is a ubiquitous immune defense strategy in which pathogen-responsive self-assembly generates cross-linked nanofibrils using nucleic acid or peptide building blocks released from host cells, resulting in the suppression of microbes. Inspired by this immune strategy, the Ee group developed β-hairpin AMPs that self-assembled into “nanonet” structures selectively in the existence of bacteria (Fig. 2 d) [34]. The expansive 3D architectures effectively trapped, detected, and killed bacteria by the antibacterial mechanism, demonstrating their potential to be used as bacteria sensors and inhibitors.

Using α-helical coiled-coil as a core structure, Lim et al. developed a supramolecular and multivalent biosensor, which exposed bacteria-binding peptides (receptors) on the external surface and embedded environment-sensitive fluorescent molecules in the internal core structure (signal producers) [42]. During the bacteria recognition event, the multivalent interaction through multiple mutual binding sites distorted the coiled-coil-based self-assembled structure, which generated fluorescent signal with the local environmental change around the fluorescent molecules. Based on this mechanism, the fluorescent supramolecular biosensor selectively detected target bacteria at a concentration level of 10^5^ cfu/mL. In addition, the bacterial-sensing ability was retained even at 50 °C, based on the high thermal stability of the coiled-coil structure.

**Fig. 3:**
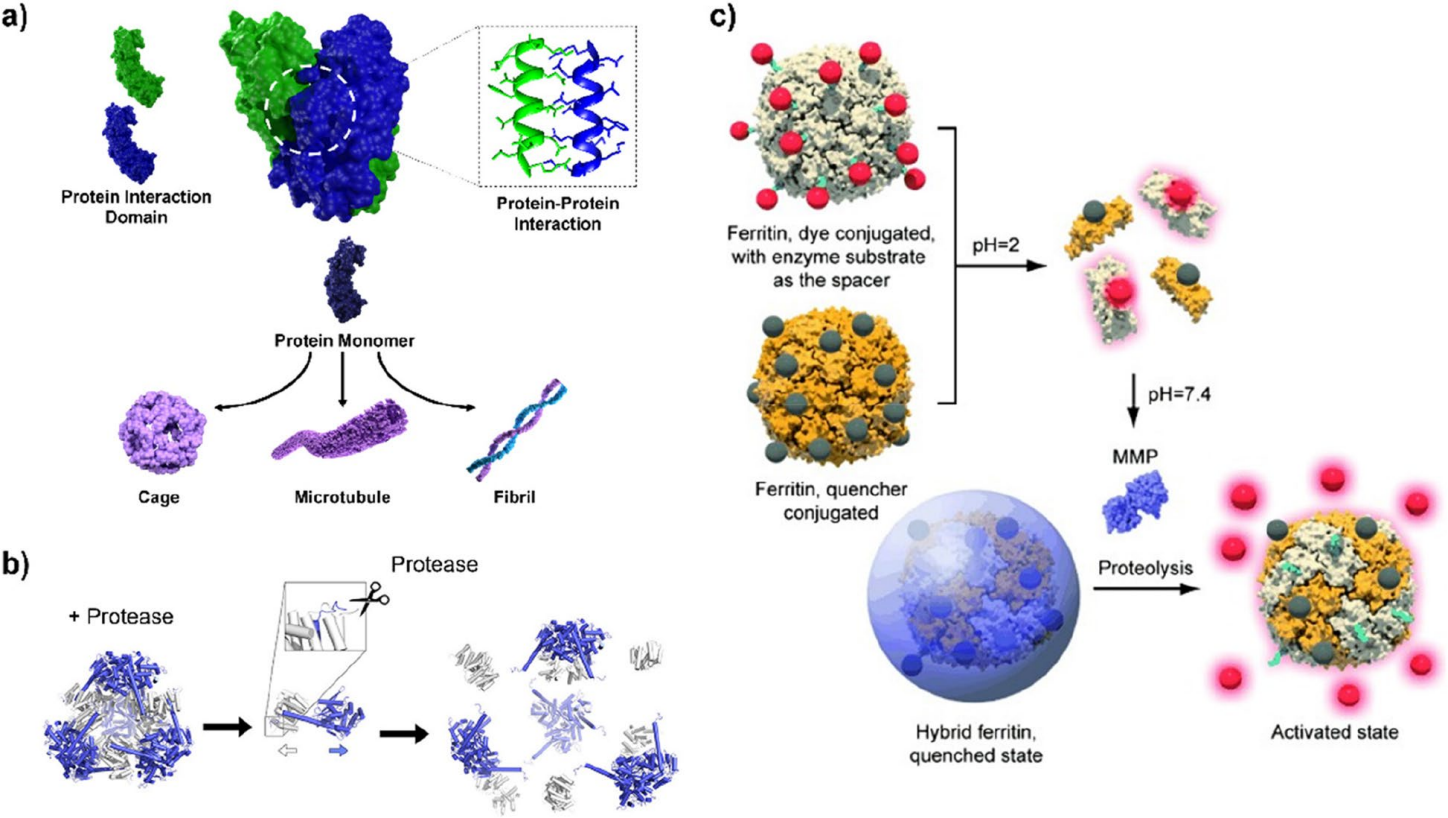
**a** Schematic illustration of self-assembly process of proteins by multiple interacting domains. **b** Schematic representation of protein nanocage system that is disassembled by highly sequence-specific protease. Reprinted with permission from ref. [52]; Copyright 2022, American Chemical Society. **c** Schematic illustration of formation of ferritin cage-based activatable probes. Reprinted with permission from ref. [53]; Copyright 2011, Wiley

Unlike peptides, proteins can establish multiple contacts with other proteins in a specific manner by displaying multiple interaction domains, thereby forming 3D structures with sophisticatedly controlled shapes and sizes (Fig. 3 a). Based on this, many self-assembling proteins organize cell organelles (e.g., filaments [43], microtubules [44], cilia [45], flagella [46], and molecular motors [47]) and facilitate intricate and integrated cellular functions such as intracellular transport, cellular motility, and cell division [48]. Utilizing such features of proteins, extensive studies have been conducted to develop artificial nano- and micro-structures that exhibit interesting biological/chemical activities, such as self-healing materials and spatially ordered multienzyme cascades [49, 50]. With the aid of artificial intelligence, it is also possible to design artificial proteins developing highly ordered 3D architectures [51].

Many self-assembling proteins can show responsive properties to various stimuli, which can be harnessed for the generation and transduction of biosensor signals. For instance, the yeast stress granule polyA-binding protein 1 (Pab1), which remained soluble in the cytoplasm at 33 °C, showed a heat-shock response of forming droplets when the temperature rose to 46 °C [54]. Proteases represent another useful signal that facilitates dynamic structural changes in proteins. The Yeates group introduced protease-cleavable sequences into proteins designed to form protein cages through self-assembly, which allowed the nanostructures to disassemble responding to the specific enzymes (Fig. 3 b) [52]. Depending on the type of introduced sequence, the protein cages exhibited selective changes in the supramolecular structure under conditions associated with diseases, such as cancer, Alzheimer’s disease, and blood coagulation. Chen et al., also developed protease-activable protein cages, which were used to produce tumor-specific fluorescent signal (Fig. 3 c) [53]. Using a metalloproteinase (MMP)-cleavable linker, molecules forming a fluorescence-quenching pair were conjugated to the ferritin proteins, which assemble into stable protein cages in the physiological condition. This design allowed the fluorophores to detach from the proteins and emit a fluorescence signal upon exposure to the protease. When the supramolecular probes were injected into a UM-SCC-22B (head and neck squamous cell carcinoma) xenograft tumor model, it was observed that the fluorescence signal was specifically and immediately generated at the tumor site and persisted for over 3 hours, which demonstrated their potential to be used as a biosensor for cancer diagnosis.

### Oligonucleotide-based supramolecular biosensors

**Fig. 4:**
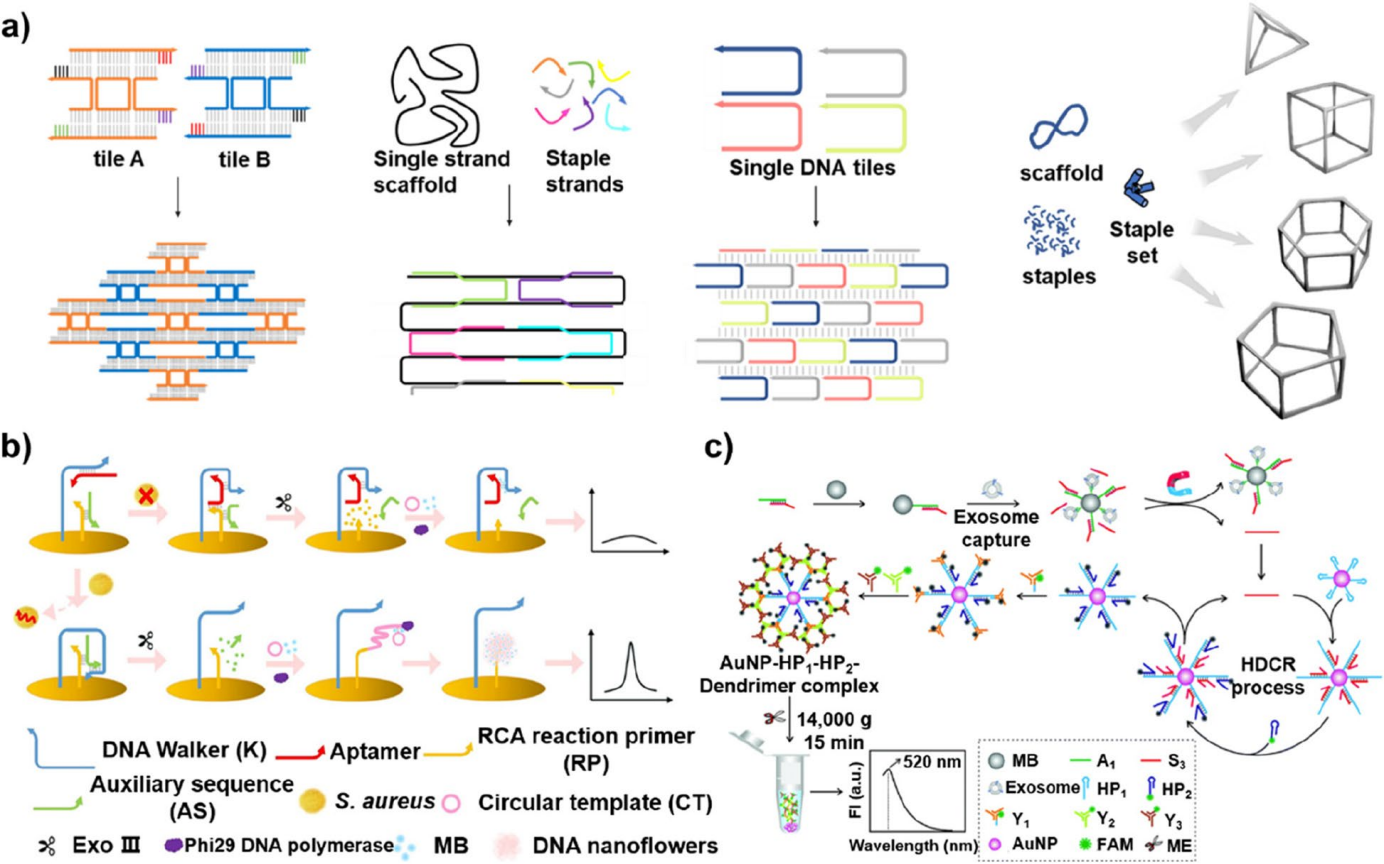
**a** Various DNA origami structures. **b** Schematic illustration of the proposed DNA walker- and DNA nanoflower-based biosensor for *S. aureus*. Reprinted with permission from ref. [67]; Copyright 2021, American Chemical Society. **c** Schematic representation of the DNA assembly-mediated dual signal amplification method for detecting exosomes. Reprinted with permission from ref. [68]; Copyright 2019, Royal Society of Chemistry

DNA molecules can fabricate nanostructures with meticulously controlled sizes, shapes, and morphologies based on specific base pairings [54–58]. By using short single-stranded DNA molecules as staples, it is possible to develop DNA tiles or bricks using long single-stranded DNA molecules, which can serve as building blocks for the construction of higher-order and complex two-dimensional (2D) arrays, 3D lattices, or polyhedral framework structures (Fig. 4 a) [59–63]. Through this process called DNA origami, many supramolecular biosensors have been designed to detect biological targets, including cells [64], circulating tumor DNA [65], and oncogenes [66]. Given that their functions are intrinsically tied to their precisely controlled 3D structures, a variety of DNA nanostructures have been utilized, such as tetrahedrons [67, 68], nanosheets [66], dendrimers [69], nanotweezer-based nanoreactors [70], and controllable nanoscale robotic arms [71].

Enterotoxins generated by *Staphylococcus aureus* (*S. aureus*), a food borne pathogen, can cause food poisoning and various infections [72]. Hence, for sensitive detection of the bacteria, the Zhou group developed a dual-signal amplification-based biosensor using a DNA walker and DNA nanoflowers [73]. As shown in Fig. 4 b, the signal generation process of the biosensor initiated with the release of *S. aureus-*binding aptamer from the DNA walker upon binding, which allowed the DNA walker to move and hydrolyze the auxiliary sequence with the assist of exonuclease III. Then, DNA nanoflowers were formed with the introduction of a circular DNA template for the rolling circle amplification (RCA) reaction and Phi29 DNA polymerase. Finally, the electroactive molecules (methylene blue, MB) interacted with the DNA duplex, increased the conductivity of DNA, and generated a strong electric signal. This approach yielded a wide dynamic range of responses that span from 60 to 6 × 10^8^ colony-forming units per milliliter (CFU/mL) and exhibited a detection limit of 9 CFU/mL.

The Ye group also employed a dual-signal amplification method for sensitive and specific detection of tumor-derived exosomes [74]. This approach was based on the complex structure of a magnetic bead substrate, CD63-binding aptamers covalently conjugated to the bead, and DNA probes bound to the aptamers (Fig. 4 c). When CD63-bearing exosomes were captured by the complex, the aptamers underwent conformational change, which resulted in the release of the DNA probes. Then, the released DNA probes can hybridize with a hairpin probe immobilized to the surface of a gold nanoparticle (AuNP), thereby triggering a catalytic hairpin DNA cascade reaction (HDCR) that serves as the initial signal amplification. Simultaneously, the open hairpin probe served as an anchor for the self-assembly process forming DNA dendrimers, which acted as secondary signal amplification. During this process, fluorescently labeled stick-ended Y-shaped DNA molecules were meticulously arranged on the surface of the AuNPs. Under conditions that are optimized for performance, this methodology has demonstrated a substantial linear response when applied to HepG2 cell-derived exosomes, across a range of concentrations spanning from 1.75 × 10^3^ to 7.0 × 10^6^ particles/μL, while retaining a detection limit of 1.16 × 10^3^ particles/μL.

**Fig. 5:**
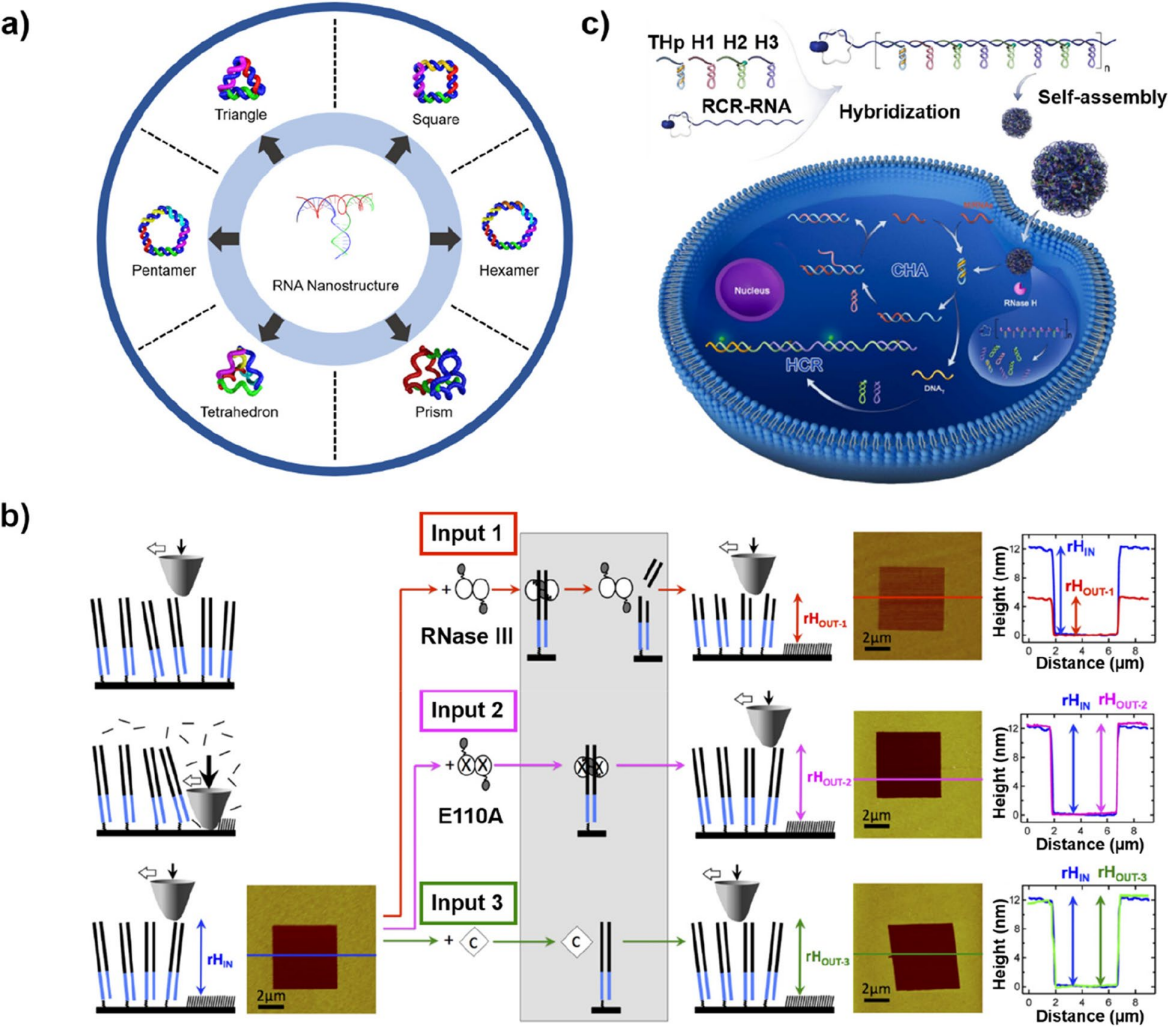
**a** Various self-assembled RNA nanostructures. **b** Illustration of the AFM-based RNA recognition and processing system based on a self-assembled ds[RNA-DNA] matrix. Reprinted with permission from ref. [85]; Copyright 2013, Springer Nature. **c** Illustration of self-assembled DNA/RNA nanospheres with cascade signal amplification for intracellular microRNA imaging. Reprinted with permission from ref. [86]; Copyright 2022, Elsevier

RNA adopts various secondary and tertiary structures using intra- and inter-molecular interactions, which are mediated by non-canonical base-pairing, base-phosphate, and base-ribose interactions, as well as Watson-Crick base-paring [75, 76]. This allows the RNA to form nanostructures with unique functional and structural properties through molecular self-assembly (Fig. 5 a) [77, 78]. In addition, the integration of RNA and DNA enables the construction of hybrid nanostructures, whereby well-defined structures can be developed into various 2D and 3D shapes, including triangles [79, 80], squares [81], cubes [82], dodecahedrons [83], and nanotubes [84].

For example, the Nicholson group developed a double-stranded DNA-RNA (ds[RNA-DNA]) chimera-based self-assembled monolayer (SAM) system for label-free detection of RNA-specific biomolecules [85]. As depicted in Fig. 5 b, they incorporated dsRNA as a probe onto a short ds[RNA-DNA]-based imprinting matrix. This biosensor system utilized the cleavage action of ribonuclease III targeting an ancillary reporter site in dsDNA, and the binding of an inactive dsRNA-binding mutant to generate a distinct digital output responding to the dsRNA-specific input. This resulted in an irreversible height change in the arrayed ds[RNA-DNA] structures, which was measured using atomic force microscopy (AFM). The alteration in height enabled the detection of interactions between the surface-exposed double-stranded RNA (dsRNA) segment and a variety of inputs, such as RNA-binding proteins, RNA-processing nucleases, and intercalating agents, without the need for labeling. Thus, by facilitating the detection and characterization of dsRNA and associated biomolecules in a restricted volume, this system offers promising potential to be used in various diagnostic genomic studies.

Xu et al. also developed DNA-RNA hybrid structures, which amplified an intracellular cascade signal for microRNA (miRNA) imaging (Fig. 5 c) [86]. They designed an RNase H-responsive self-assembled DNA/RNA nanosphere (NS) by integrating rolling cycle replication (RCR)-generated long single-stranded RNA and four types of functional DNAs. Once NSs internalized into cells, intracellular RNase H triggered the degradation of the RNA in the DNA/RNA complex, releasing DNAs designed to specifically recognize the target miRNA, along with additional DNAs acting as indicator probes. Upon interaction with the target miRNA, successive double-cycle amplification of catalytic hairpin assembly (CHA) and hybridization chain reaction (HCR) improved the detection sensitivity of the sensing platform through the restoration of quenched fluorescence signal. This approach led to the successful detection of low-expressed miRNA 155 in MCF-7 and HeLa cells. This platform could screen out abnormal cells based on the abundance of miRNAs and has versatility in its potential application to different target miRNAs via simple modification. Consequently, it offers the potential for early disease diagnosis utilizing miRNA-based approaches.

## Electrochemical biosensing platforms

### Voltammetric and amperometric biosensors

**Fig. 6:**
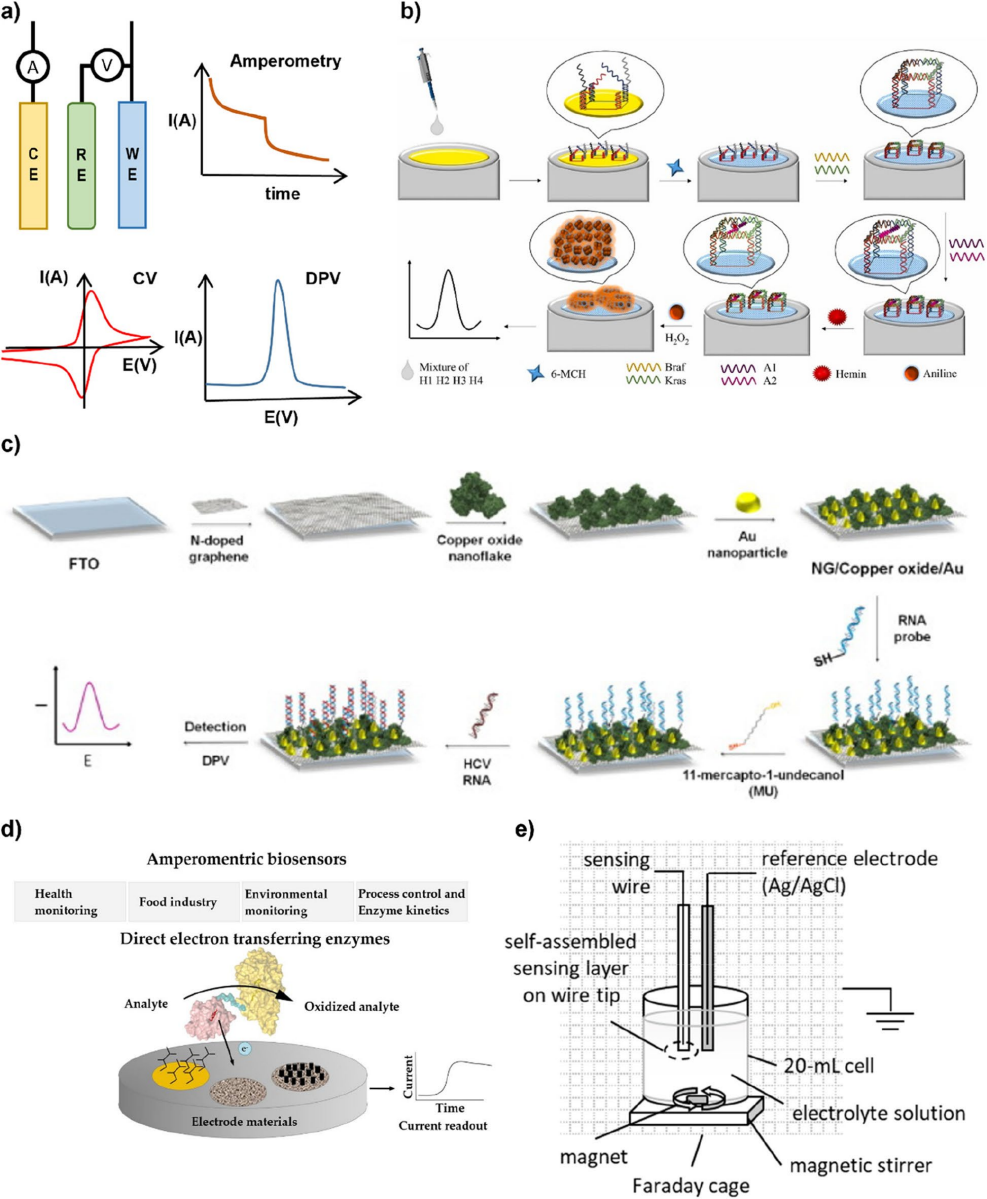
**a** Diagram of a voltammetric/amperometric biosensor consisting of three types of electrodes: working electrode (WE), reference electrode (RE), and counter electrode (CE), and typical plots of the resulting signals for amperometry, cyclic voltammetry (CV), and differential pulse voltammetry (DPV). **b** The self-assembly of single-stranded probes, target DNAs and assistant probes to DHN nanostructure on the proposed electrochemical biosensor. Reprinted with permission from ref. [91]; Copyright 2021, Elsevier. **c** Functionalizing process of RNA for HCV detection based on gold nanoparticles-coated FTOE and target detection using DPV. **d** Schematic illustration of the metabolite-detecting amperometric biosensors. **e** Electrochemical set-up for metal-supported self-assembled lipid membrane biosensor for detecting phenol in water. Reprinted with permission from ref. [92]; Copyright 2021, Elsevier

In voltammetry, a time-dependent potential is administered to an electrochemical cell, and the resulting current is assessed in relation to the applied potential, while amperometry involves the application of a consistent potential to the working electrode, with the subsequent current being monitored in relation to time [87]. Voltammetric and amperometric biosensors function within a triad of electrodes, comprising a working electrode responsible for target recognition, a counter electrode serving as a current source, and a reference electrode to maintain a consistent potential (Fig. 6 a) [88]. The current signal arises through an electrochemical reaction at the working electrode, investigated by the applied potential, facilitating accurate target quantification [89]. The voltammetric biosensors can be performed through various methods, including cyclic voltammetry, differential pulse voltammetry, and square wave voltammetry [90].

Affinity recognition is indispensable for the detection of disease biomarkers, including proteins and nucleic acids, with most biosensors adopting this approach falling under the category of voltammetric biosensors [93, 94]. Since these sensors require electroactive labeling for target detection, they can be fabricated by immobilizing a capture biomolecule (e.g., DNA, peptides, antibodies, aptamers) on a working electrode to detect proteins or nucleic acids using a sandwich assay format, and self-assembling molecules described in the previous section can be used.

For example, Zhou et al. developed a size-tunable multifunctional DNA hexahedral nanostructure (DHN) for detecting mutations in Kras and Braf DNA (oncogenes in ovarian carcinoma) (Fig. 6 b) [91]. The DHN including four single-stranded DNA probes (H1, H2, H3, and H4), which were designed by computer simulation, was immobilized on a gold electrode via the adsorption of polyadenines (polyA) on the gold surface. Along with the interactions with target DNAs and assistant probes, DHN formed the complete DNA nanostructure and catalyzed aniline polymerization, which produced intensive electrochemical signal. Based on this mechanism, the DHN-based biosensor achieved specific detection of target DNA and their mutations at femtomolar concentration level.

Omidinia E et al. reported the development of a label-free and ultrasensitive nanobiosensor for the Hepatitis C virus (HCV) detection (Fig. 6 c) [95]. The biosensor was based on a fluorine-doped tin oxide electrode (FTOE) coated by gold nanoparticles, which was functionalized with thiolated complementary probes of the RNA virus through the SAM process. Differential pulse voltammetry (DPV) using Fe(CN)_6_^3−/4-^ as a redox probe revealed a significant change in response to interactions of the target RNA of the HCV virus with the complementary sequence. The experimental results yielded a remarkably broad range of linearity, ranging from 1 × 10^−15^ to 1 × 10^−6^. Furthermore, the sensor demonstrated exceptional sensitivity by detecting ultralow concentrations of viral RNA with a detection limit of 1 × 10^−15^.

On the other hand, amperometric biosensors are the most widely used sensors for metabolite detection [96]. In this type of biosensors, a target-specific enzyme is affixed to a working electrode, thereby initiating the oxidation of the target substance under a consistent potential [97]. These metabolite-detecting amperometric biosensors are relatively simple to fabricate and have the advantage of high sensitivity and selectivity because they detect metabolites by enzyme recognition, which results in direct electron transfer (Fig. 6 d) [98].

Georgopoulos et al. reported a metal-supported self-assembled lipid membrane biosensor for detecting pollutants in water, such as horseradish peroxidase and phenol in water (Fig. 6 e) [92, 99]. The electrochemical setup utilized for the metal-assisted peroxide biosensor comprised a two-electrode configuration, which was assembled to include a sensing wire that hosted a reference Ag/AgCl electrode and the enzyme/lipid membrane complex on its tip. Both electrodes were submerged in an electrolyte solution. The sensitivity of the metal-supported peroxide biosensor was found to be 1.4071 nA/μM of hydrogen peroxide solution, and the limit of detection was determined to be 0.083 μM. The biosensor was able to reliably detect the lowest peroxide concentration in several water samples from tap, lake, and river, which was 0.95 mM. On the other hand, the phenol sensor exhibited the detection limit, determined by a signal-to-noise ratio of 3, was 1.24 pg/mL, while the sensitivity was found to be 33.45 nA per pg/mL of phenol concentration. Further validation tests using real water samples revealed that the sensor could detect phenol at concentrations as low as 6.1 ppb in lake water and 2.5 ppb in tap and river water without requiring any pretreatment. Therefore, it was demonstrated that lipid bilayers, whether metal-supported or freely suspended, can be utilized for monitoring association-dissociation events and molecular aggregation phenomena on their surfaces.

### Potentiometric biosensors

**Fig. 7:**
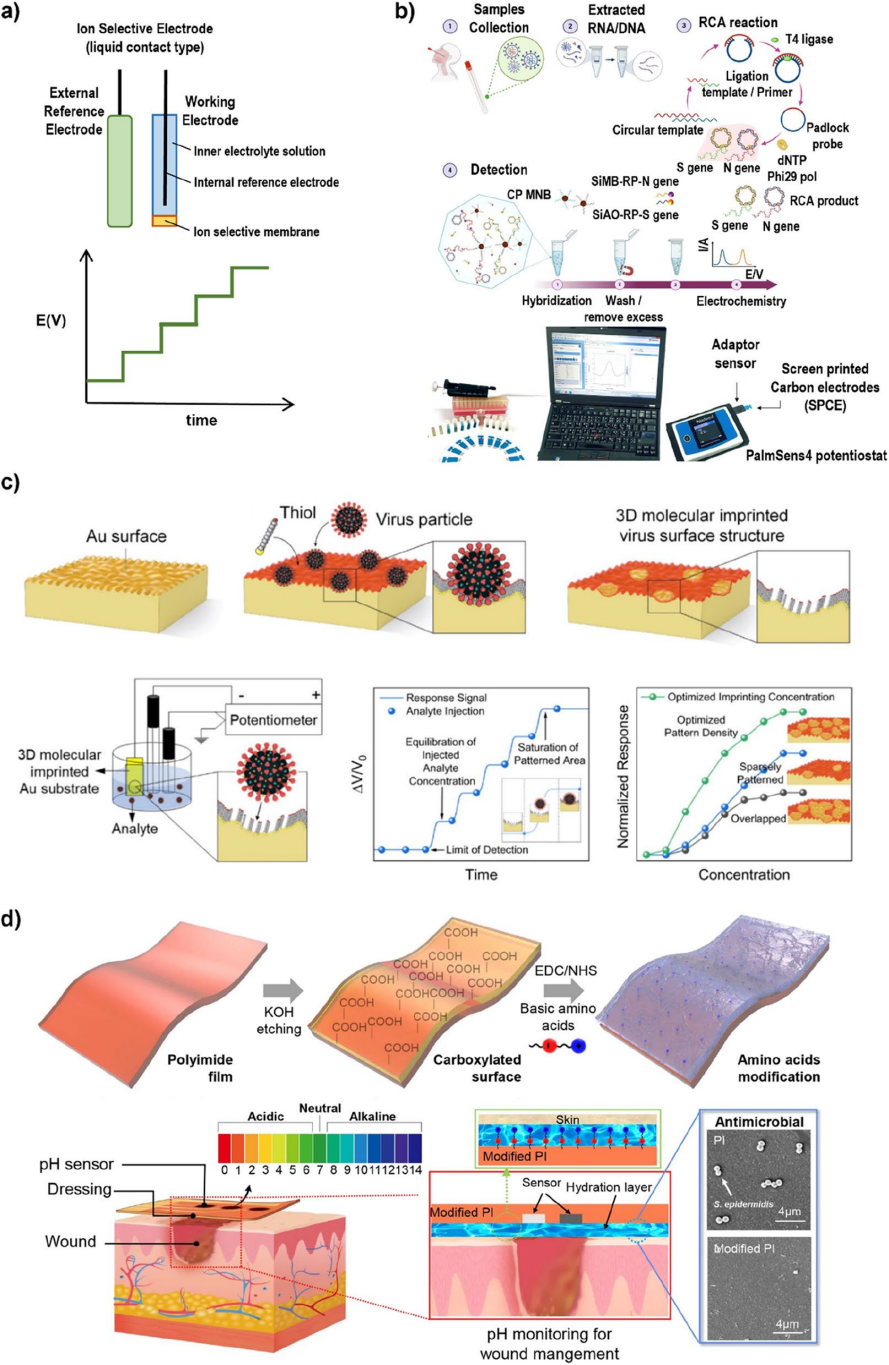
**a** Potentiometric biosensor based on an ion-selective electrode and a typical plot of the resulting signal. **b** Detection workflow of SARS-CoV-2 from clinical samples using the electrochemical biosensor and the detection setup for electrochemical analysis. Reprinted with permission from ref. [102]; Copyright 2021, Springer Nature. **c** The 3D molecular imprinting process and potentiometric sensing data plots for virus detection. Reprinted with permission from ref. [103]; Copyright 2022, American Chemical Society. **d** Schematic of preparation, mechanisms, and design of pH-monitorable wound healing dressing with antimicrobial activity. Reprinted with permission from ref. [104]; Copyright 2023, Springer Nature

A potentiometric biosensor represents a class of electrochemical biosensors designed to detect and quantify analytes by measuring the potential difference or voltage generated during a specific chemical reaction that involves the target molecule [17]. This class of biosensors operates on the premise that certain chemical reactions induce changes in ion concentrations, altering the electrical potential at the electrode-solution interface [100]. Potentiometric biosensors typically operate in a two-electrode system: a working electrode and a reference electrode (Fig. 7 a) [88]. The working electrode is designed to selectively interact with the target analyte or specific ion, and when the analyte of interest interacts with the surface of the working electrode, it causes a change in the local ion concentration, which in turn changes the potential at the electrode-solution interface [101]. This potential change is then measured by comparing it to the stable potential of the reference electrode.

The main advantages of potentiometric biosensors are fast response time and relatively low cost [105]. This type is suitable for detecting ions, gases, and molecules that can induce ion concentration changes, and is particularly often used in applications requiring precise pH measurement and ion concentration measurement [106, 107]. Ion-selective electrodes (ISEs) and ion-sensitive field-effect transistors (ISFETs), designed to be selectively sensitive to specific ions based on membrane composition, are common types of potentiometric biosensors [108, 109]. Ion-selective membranes allow only target ions to permeate, resulting in a potential change on the electrode surface [110, 111].

Recently, the rapid electrochemical detection of SARS-CoV-2 virus using the isothermal rolling cycle amplification (RCA) technique has been demonstrated due to the simplicity inherent in potentiometric detection (Fig. 7 b) [102]. Rafailovich et al. also showcased the potentiometric sensor using three-dimensional (3D) molecular imprinting as an effective method for viral testing (Fig. 7 c) [103]. In this study, it was proved that two different subtypes of influenza A virions, H1N1 and H3N2, and the purified S-proteins of SARS-CoV-2 and middle east respiratory syndrome (MERS) viruses, could be detected in human saliva, respectively. The biosensor provided the sensitivity of RT-PCR in detecting very low viral titers and differentiating different viral subtypes while producing results in less than 5 min.

Moon et al. developed a wound-healing dressing film containing basic amino acid-modified polyimide and capable of pH monitoring by potentiometric methods (Fig. 7 d) [104]. The basic amino acids (Lys, Arg) provided the polyimide surface with cationic functional groups and promoted antibacterial activity, exhibiting antibiofilm activity analogous to cationic antimicrobial peptides. They also showed that the pH sensor in the form of polyimide biofilms functionalized with basic amino acids worked well under different pH conditions and bacterial infection levels on human skin. These findings are expected to contribute to the field of wearable healthcare devices due to the advantages of potentiometric biosensors.

### Impedimetric biosensors

**Fig. 8:**
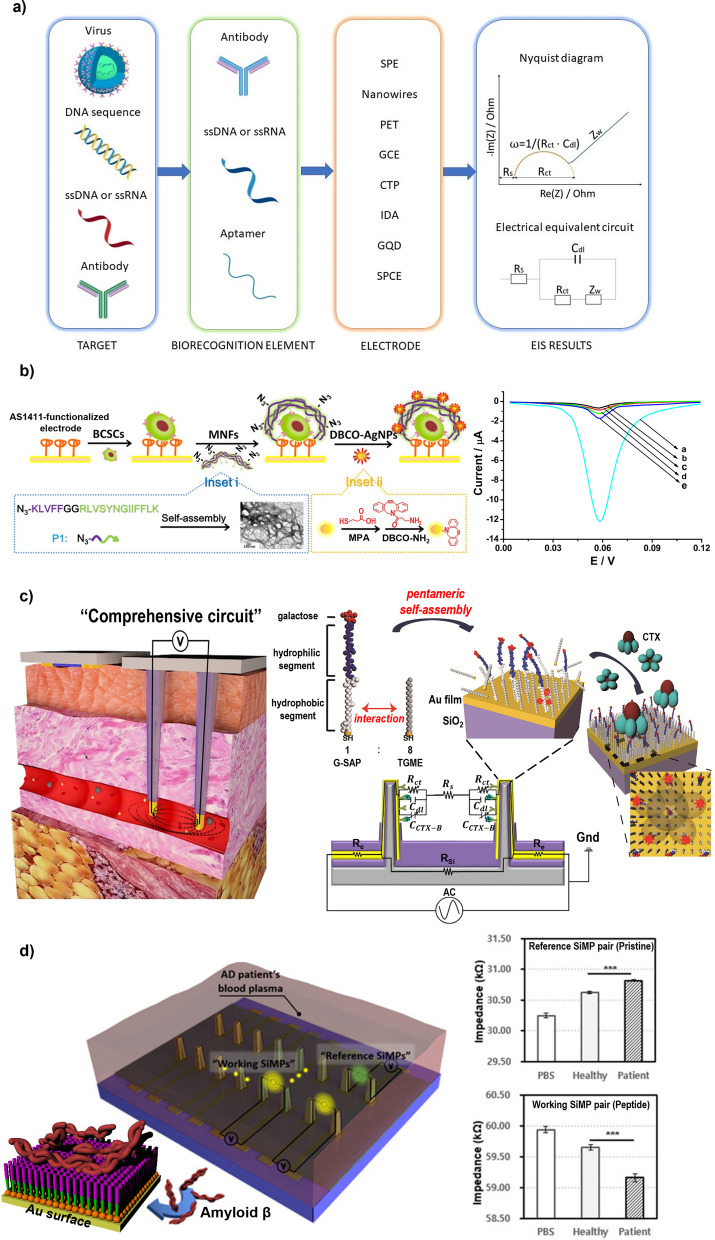
**a** Components of an impedimetric biosensor and the equivalent electric circuits (EEC) and Nyquist diagram for interpreting electrochemical impedance spectroscopy (EIS) data. **b** Electrochemical identification of BCSCs on electrodes using multifunctional nanofibers and data plot of results showing detection of BCSCs targeted by a surface-functionalized electrode. Curve a indicates the presence of BCSCs. Reprinted with permission from ref. [117]; Copyright 2019, American Chemical Society. **c** Silicon micropillar arrays functionalized with self-assembling peptides to form comprehensive circuits in blood vessels and the circuit diagram of a 3D biosensor for impedimetric sensing. (*R*_*e*_: Electrode resistance, *R*_*s*_: Solution resistance, *R*_*si*_: Substrate resistance, *R*_*ct*_: Charge transfer resistance, *C*_*dl*_: Double layer capacitance, *C*_*CTX* − *B*_: Capacitance of CTX-B). Reprinted with permission from ref. [118]; Copyright 2019, American Chemical Society. **d** Schematic image of the 3D sensor constructed with working and reference SiMP pairs (Pristine & Peptide) in the blood plasma system [119]

An impedimetric biosensor is a type of electrochemical biosensor designed to detect and quantify analytes by measuring the change in electrical impedance that occurs when a target molecule interacts with the biorecognition element on the surface of the sensor (Fig. 8 a) [112]. This biosensor is based on the principle that the presence of an analyte changes the conductivity of a solution near the sensor’s electrode surface [113]. Impedance biosensors typically consist of a working electrode and a reference electrode [114]. The working electrode is functionalized with a biorecognition element that selectively interacts with the target analyte. When the analyte binds to the biorecognition element on the surface of the working electrode, it causes a change in the local electrical properties of the surrounding solution, resulting in a measurable change in impedance [115, 116].

Impedance is essentially a measure of the resistance to alternating current (AC) flow in an electrical circuit [120]. Within an impedance biosensor, a change in impedance is caused by a change in the dielectric properties of the solution due to the presence of the analyte [121]. This change in impedance can be quantified by applying an AC signal to the biosensor and measuring the resulting changes in current and voltage across the electrodes [122].

One of the notable advantages of impedimetric biosensors is their label-free nature [122]. Unlike some other biosensors that require labeling or chemical modification of the target molecule, impedimetric biosensors directly measure the change in electrical properties induced by the interaction between the biorecognition element and the analyte [123]. This property can simplify the analytical process and lead to faster and more straightforward measurements.

Impedimetric biosensors are therefore applicable in a variety of fields, including medical diagnostics, environmental monitoring, and food safety [124, 125]. They are particularly well suited for real-time monitoring and interaction involving larger biomolecules such as cells, proteins, and nucleic acids [113, 126]. Although impedimetric biosensors have been greatly improved during the COVID-19 pandemic, their practical implementation often suffers non-specific binding to non-target compounds, reducing selectivity and sensitivity [88], so elaborated design to increase selectivity is critical in the application of self-assembling biomolecules.

For instance, Zhao et al. demonstrated a sensitive electrochemical method for the identification of the stemness marker CD44, which is present at extremely low levels in body fluids, in breast cancer cells (BCSCs) (Fig. 8 b) [117]. This was achieved via the use of self-assembling peptide-based multifunctional nanofibers (MNFs), which were designed to bind to stem-like cells using CD44-binding peptides and to recruit DBCO-functionalized silver nanoparticles (AgNPs) using N_3_ groups for the generation of electrochemical signals. As the nanofiber provided many reaction sites, multiple AgNPs were recruited and exhibited significantly amplified electrochemical signals. The responses gradually increased with the addition of different concentrations of BCSCs with the limit of detection (LOD) of 6 cells/mL (defined as a signal-to-noise ratio of three) within a wide linear range of 10 to 5 × 10^5^ cells/mL.

Choi et al. engineered a 3D biosensor, utilizing a silicon micropillar array (SiMPA) electrodes and self-assembling peptides to detect targets through impedance alterations (Fig. 8 c) [118]. This SiMPA biosensor forms a comprehensive circuit that includes blood, electrodes, and receptors as circuit components. It accomplishes target detection by monitoring impedance changes resulting from the interaction of peptides with protein receptors like cholera toxin or heavy metal ions, such as mercury ions, on the SiMPA electrode surface. The research authenticated the sensor’s mechanism, establishing its capability for real-time, high-sensitivity, and selective target detection in blood, even under the intricate conditions inherent to the blood system.

They also applied the biosensor to detect amyloid beta protein, confirming the versatility of the biosensor by impedimetric method (Fig. 8 d) [119]. Detecting amyloid beta (Aβ) in the blood is quite difficult due to its low concentration and the presence of other substances. Lim and Choi et al. proposed a 3D sensor that can detect Aβ in blood by using 3D silicon micropillar (SiMP) electrodes surface-treated with peptides that bind to Aβ and a spatial circuit configuration. The 3D SiMP comprehensive circuit was able to detect the target protein in blood more effectively than 2D electrodes due to its structural advantages, large exposure area, and impedimetric analysis system. The highly sensitive 3D sensor showed high accuracy even at low Aβ concentrations in the blood plasma of AD patients. These results suggest the potential of an impedimetric 3D biosensor for AD diagnosis.

Liu et al. reported a label-free biosensor, employing self-assembled RNA riboswitch and electrochemical impedance spectroscopy (EIS), specifically designed for the sensitive and selective detection of cyclic diguanylate monophosphate (c-di-GMP) – an allosteric regulator of cellulose synthesis in Acetobacter Xylinum [127]. The electrochemical behavior of the c-di-GMP/riboswitch/Au electrode measured by differential pulse voltammetry (DPV) during the biosensor system assembly demonstrated that variations in the electrostatic interactions environment was induced by the binding of c-di-GMP to the nucleic acid receptors. This riboswitch-EIS-based biosensor revealed a linear detection range between 50 and 1000 nM, along with a noteworthy low detection limit of 50 nM. Evaluative experiments highlighted the biosensor’s selective recognition capabilities; a substantial impedance signal increase was noted in the presence of c-di-GMP, while negligible impedance variations were observed with cAMP, GTP, ATP, or AMP. Thus, this impedimetric biosensor demonstrated that nanoscale-structured RNA can be used for sensitive and selective detection of target molecules.

## Conclusions and outlook

As described above, a number of BSNs have been developed for biosensor applications based on the unique structural characteristics of supramolecules. Despite their potential, the more widespread use of BSN-based biosensors has been limited for several reasons. First, it is generally difficult to develop nanostructures with precisely controlled sizes and shapes through self-assembly because direct control of the spontaneous molecular behavior is impossible. Second, the large-scale preparation of uniform nanostructures without nonspecific aggregation is highly challenging. Third, the influence of the in vivo environment and substances on the physicochemical properties of self-assembled nanostructures is not yet fully understood.

These problems are expected to be resolved as technological development progresses and the understanding of the use of nanomaterials in biological systems is intensified. In addition, there are a variety of electrochemical platforms applicable to biosensing, including the voltammetric, amperometric, potentiometric, and impedimetric sensors mentioned above, so it is important to understand them and apply the appropriate technology for target molecules detection. On the other hand, since self-assembled nanostructures have the potential to be used not only as biosensors but also as drug delivery vehicles and therapeutic agents, there will likely be an increasing interest in developing BSN-based biosensors that can exhibit additional therapeutic functions. In addition, the types of target substances for BSN-based biosensors could expand beyond biomaterials, along with advances in library screening techniques. Therefore, BSN are expected to be mainstream platforms in the field of biosensor development.

## Data Availability

Not applicable.

## Abbreviations

BSNs: Biomolecule-based self-assembled nanostructures
SPPS: Solid-phase peptide synthesis
ALP: Alkaline phosphatase
SAM: Self-assembled monolayer
SAM-P: Self-assembling monomer precursor
NBD: Nitrobenzoxadiazole
AMP: Antimicrobial peptides
Pab1: PolyA-binding protein 1
MMP: Metalloproteinase
RCA: Rolling circle amplification
RCR: Rolling cycle replication
AuNP: Gold nanoparticle
HDCR: Hairpin DNA cascade reaction
AFM: Atomic force microscopy
CHA: Catalytic hairpin assembly
HCR: Hybridization chain reaction
DHN: DNA hexahedral nanostructure
PolyA: Polyadenines
HCV: Hepatitis C virus
FTOE: Fluorine-doped tin oxide electrode
DPV: Differential pulse voltammetry
ISEs: Ion-selective electrodes
ISFETs: Ion-sensitive field-effect transistors
MERS: Middle east respiratory syndrome
AC: Alternating current
BCSCs: breast cancer cells
MNFs: Multifunctional nanofibers
AgNPs: Silver nanoparticles
LOD: Limit of detection
SiMPA: Silicon micropillar array
EIS: Electrochemical impedance spectroscopy
C-di-GMP: Cyclic diguanylate monophosphate
